# Reciprocal Effects Among Parental Homework Support, Effort, and Achievement? An Empirical Investigation

**DOI:** 10.3389/fpsyg.2018.02334

**Published:** 2018-11-30

**Authors:** Jianzhong Xu, Jianxia Du, Shengtian Wu, Hailey Ripple, Amanda Cosgriff

**Affiliations:** ^1^Department of Counseling, Educational Psychology, and Foundations, Mississippi State University, Starkville, MS, United States; ^2^Faculty of Education, University of Macau, Taipa, Macau

**Keywords:** achievement, autonomy, effort, parental homework support, parent involvement

## Abstract

The present study investigates reciprocal influences of parental homework support, effort, and math achievement, using two waves of data from 336 9th-graders. Results revealed that higher prior autonomy-oriented support and homework effort resulted in higher subsequent achievement. Higher prior content-oriented support led to higher subsequent effort, but lower subsequent achievement. Additionally, higher prior effort led to higher subsequent autonomy-oriented support. Furthermore, our results supported the structural path invariance over gender. The current investigation advances extant research, by differentiating two forms of parental homework support (autonomy- and content-oriented support), and by showing their respective influences on subsequent homework effort and math achievement.

## Introduction

Parent involvement in homework has garnered much attention from educators and policy makers (Patall et al., 2008; Moroni et al., 2015), as there are generally consistent findings that homework has a positive effect on student achievement (Cooper et al., 2006; Fan et al., 2017), and as homework has everyday importance for teachers, parents, and students (Cooper et al., 2006). Thus, it is not surprising that parental homework involvement is viewed as an important strategy to promote student achievement (Hoover-Dempsey et al., 2001; Patall et al., 2008; Dumont et al., 2012) and desirable attributes (e.g., effort and self-regulation; Xu and Corno, 1998; Ramdass and Zimmerman, 2011).

Research on parental homework involvement, however, has yielded inconsistent findings for several reasons (Pomerantz et al., 2007; Patall et al., 2008; Hill and Tyson, 2009; Moroni et al., 2015). First, although parental homework involvement takes different forms (e.g., parental control and direct aid; Patall et al., 2008), previous research has not paid adequate attention to “multidimensional measures in order to come to consistent conclusions about the effectiveness of parental involvement in homework” (Moroni et al., 2015, p. 418). Second, recent literature taps into one promising form of parent involvement – parental support in homework (Dumont et al., 2014; Moroni et al., 2015; Silinskas and Kikas, 2017). However, it has not differentiated two forms of parental support in homework: (a) autonomy-oriented support (i.e., paying attention to children’s ideas and encouraging their homework initiatives), and (b) content-oriented support (i.e., offering direct help on homework assignments). Finally, except for several exceptions (e.g., Dumont et al., 2014; Moroni et al., 2015), previous research relied on cross-sectional data, thereby unable to disentangle the direction of relation between parent involvement and student outcome.

To address these limitations in prior research, we examine the temporal ordering of parental homework support (including both autonomy- and content-oriented support), effort, and achievement, using two waves of data from 9th-graders.

### Theoretical Framework

One framework pertaining to parental homework involvement is self-determination theory (Deci and Ryan, 2008; Ryan et al., 2016). Self-determination theory postulates that the needs for autonomy, competence, and relatedness are “essential for facilitating optimal functioning of the natural propensities for growth and integration, as well as for constructive social development and personal well-being” (Ryan and Deci, 2000, p. 68). The need for autonomy concerns the feelings of volition that accompanies an activity (e.g., having the freedom to act, feel, or think for themselves). The need for competence concerns experiences of mastery in carrying out an activity (e.g., having a sense of proficiency). The need for relatedness concerns having trustful and warm relationships (e.g., feeling connected with important others). As “contexts supportive of autonomy, competence, and relatedness were found to foster greater internalization and integration than contexts that thwart satisfaction of these needs,” Ryan and Deci (2000) argued, it was of “great significance for individuals who wish to motivate others in a way that engenders commitment, effort, and high-quality performance” (p. 76). Specifically, autonomy support from significant others (e.g., parents) can foster children’s need satisfaction, by nurturing their volitional functioning, by taking an active interest in their frame of reference, and by encouraging them to take personal initiative (Ryan et al., 2016).

Closely related to self-determination theory, Grolnick and Slowiaczek (1994) conceptualized two models concerning the effects of parent involvement: a direct effect and an indirect effect. The direct effect model posits that parent involvement affects students’ schooling through directly teaching them relevant academic skills (e.g., providing direct assistance on homework assignments). The indirect effect model posits that parent involvement affects students’ schooling through indirectly fostering their motivation to do well in school (e.g., providing autonomy support and encouraging children to put forth effort in homework assignments). Based on their review of related studies concerning these two models, Raftery et al. (2012) found that “parent involvement may likely have its largest effects by facilitating the attitudes and values children need to put forth effort in school” (p. 348).

Grolnick and Slowiaczek (1994) further hypothesized that associations between parent involvement and student achievement may be reciprocal: “While the parent-to-child effects model may be plausible, equally plausible is the model whereby parent involvement follows student competencies” (p. 240). Based on recent studies relating to parental autonomy support and assistance (Pomerantz and Eaton, 2001; Bronstein et al., 2005), Raftery et al. (2012) similarly posited that associations between parent involvement and student outcome “may represent a bidirectional effect” (p. 348) – “parents may have higher expectations for their high-performing students” (p. 348) and “autonomy support affects motivation, motivation results in engagement, and engagement feeds back to motivational processes and parenting” (p. 352).

### Parental Homework Support, Effort, and Achievement

Several studies has investigated relations between several forms of parental homework involvement and achievement (Dumont et al., 2014; Moroni et al., 2015; Xu et al., 2017). Moroni et al. (2015) examined the impacts of parental homework involvement on reading achievement, based on 1,685 6th graders from Switzerland. Parent involvement was assessed in two forms: (a) involvement perceived as supportive, and (b) involvement perceived as intrusive. Results revealed that student achievement was positively associated with supportive involvement, but negatively related to intrusive involvement. As supportive involvement was positively associated with student achievement (after controlling prior achievement and family background), it would be important to pay more close attention to the construct of parental homework support. A close look at the 5-item scale on supportive involvement in Moroni et al. (2015) revealed that several items measured content support (e.g., “I can ask my parents any time if I don’t understand my German homework”), relating to Grolnick and Slowiaczek (1994) direct effect model. Meanwhile, other items measured autonomy support (e.g., “When my parents help me with my homework, they always encourage me first to find the correct answers for myself”), relating to Grolnick and Slowiaczek (1994) indirect effect model.

Similarly, Dumont et al. (2014) used a 4-item scale labeled as perceived parental responsiveness, in which some items assessed content support (e.g., “When I’m doing my homework, I can ask my parents for help at any time”), while others assessed autonomy support (e.g., “When I’m doing my homework, my parents carefully listen to how I would solve a problem instead of telling me what to do”).

To examine whether autonomy- and content-oriented support are empirically distinguishable, Xu et al. (2017) validated the Parental Homework Support Scale (PHSS) based on 796 8th graders in China. Both EFA and CFA results revealed that the PHSS included two subscales: Autonomy-oriented Support (4-item; α = 0.91) and Content-oriented Support (4-item; α = 0.88). Additionally, in line with theoretical expectations, the PHSS was positively associated with motivational beliefs, homework completion, and homework grade. Meanwhile, math achievement was positively associated with autonomy-oriented support, yet unrelated to content-oriented support. These findings imply the need to differentiate autonomy support from content support in research on parental homework support.

In addition to student achievement, it is important to incorporate student effort in research on parent involvement, as self-determination theory emphasizes “the great significance” to motivate individuals “in a way that engenders commitment, effort, and high-quality performance” (Ryan and Deci, 2000, p. 76). As one important goal of homework is to promote children’ ability to take responsibility for their own learning (Ramdass and Zimmerman, 2011; Dumont et al., 2014), homework effort has been conceptualized as an important construct and outcome variable (Trautwein et al., 2006; Dumont et al., 2014). Using 1,501 8th graders in Swiss in the domain of French as a foreign language, Trautwein et al. (2006) linked homework effort to two forms of involvement: (a) parental provision of help (e.g., “My parents help me with French if I ask them.”), and (b) unwanted parental help (e.g., “My parents sometimes help me with French even when I don’t need any help at all.”). Their study found that homework effort was positively related to parental provision of help, yet unrelated to unwanted parental help.

Using 2,820 German students in grade 5 and grade 7, Dumont et al. (2014) investigated reciprocal relations among parental homework involvement, reading achievement, and academic functioning (reading effort and homework procrastination). Parental homework involvement was conceptualized in three forms: (a) perceived parental control (e.g., “My parents help me with my homework even when I don’t need any help.”), (b) perceived parental responsiveness (e.g., “My parents help me with my homework if I ask them to.”), and (c) perceived parental structure (e.g., “My parents make sure that I have enough time and space to do my homework.”). Prior parental structure positively influenced subsequent reading effort, while prior reading effort positively influenced parental control and parental structure. Additionally, prior reaching achievement negatively influenced subsequent parental control. Yet, prior parental homework involvement (control, responsiveness, and structure) did not influence subsequent reading achievement.

In summary, this body of literature suggests possible associations among parental homework involvement, effort, and achievement. It points to the importance of focusing on parental homework support (instead of parental homework involvement in general), as there is more conclusive evidence in prior studies that one dimension of parental homework involvement was negatively related to effort and achievement, whether labeled as intrusive involvement (Moroni et al., 2015), parental control (Silinskas and Kikas, 2017; Dumont et al., 2014), or unwanted parental help (Trautwein et al., 2006). Additionally, it points to the importance of differentiating autonomy-oriented support from content-oriented support, as items for autonomy- and content-oriented support were often combined in one scale in prior studies, whether labeled as supportive involvement (Moroni et al., 2015), perceived parental responsiveness (Dumont et al., 2014), or perceived parental support (Silinskas and Kikas, 2017).

### The Current Investigation

The goal of our current investigation is to examine reciprocal effects among autonomy- and content-oriented support, effort, and math achievement. Specifically, it employs models of reciprocal effects, along with invariance tests across gender. This line of research is important, as parents’ behavior (e.g., homework support) may influence the child’s behavior, and as the characteristics of the child (e.g., prior achievement and effort) may also affect parents’ behavior (e.g., homework support; Grolnick and Slowiaczek, 1994; Raftery et al., 2012; Dumont et al., 2014; Kikas and Silinskas, 2016). Compared with cross-sectional models, reciprocal effects models are especially useful for examining relationships among variables over time (e.g., regarding theorized directions of influences; Little, 2013; Newsom, 2015).

Our reason for examining parental support in math homework is that parental homework involvement may differ based on subject matter, yet prior research tends to examine parental homework involvement in general (Silinskas and Kikas, 2017). In addition, students often invest significant time on math homework (e.g., 20–40% of homework time; Xu, 2017). Moreover, doing math homework often presents a significant challenge for many children and their parents (Else-Quest et al., 2008).

The reason for testing invariance across gender is that the forms of parent involvement may be different for boys and girls (e.g., parental support; Grolnick and Slowiaczek, 1994; Dumont et al., 2012; Silinskas and Kikas, 2017). Additionally, prior research on gender differences in math has yielded inconsistent results (Halpern et al., 2007; Dumont et al., 2012; Silinskas and Kikas, 2017). Furthermore, prior studies have shown mixed findings concerning gender differences in the relations between parent involvement and student achievement (Pomerantz et al., 2007; Silinskas et al., 2013). Thus, it is important to study whether relations among parental homework support, effort, and math achievement vary by gender.

### Hypotheses

#### Hypothesis 1 (Path Coefficients)

**FIGURE 1:**
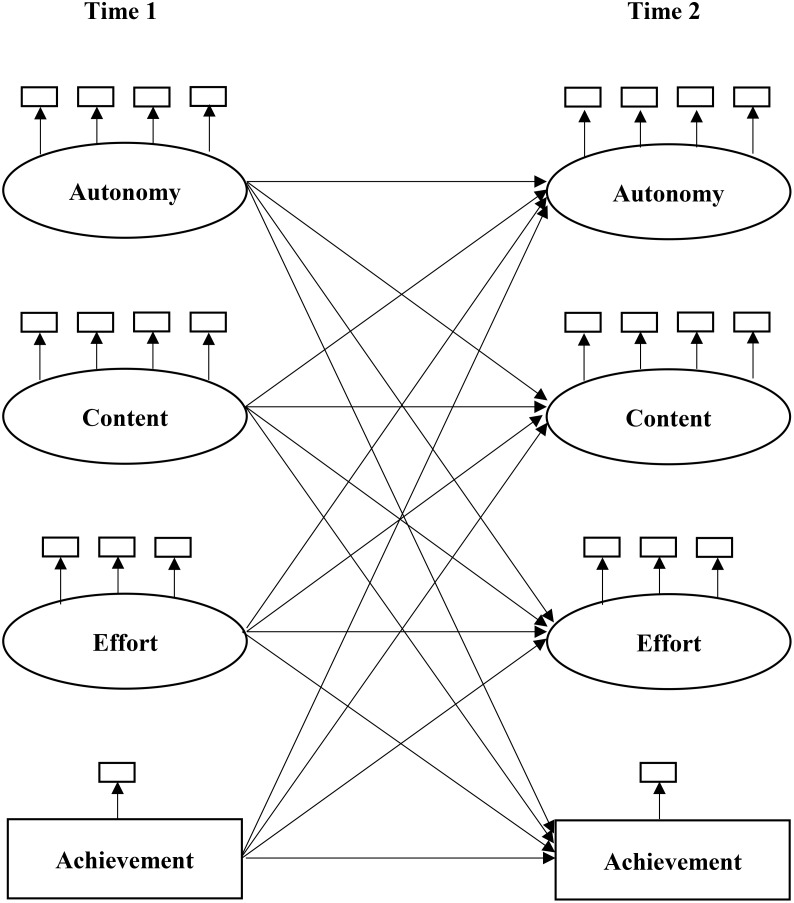
The model of reciprocal effects among autonomy-oriented support, content-oriented support, effort, and achievement.

Models of reciprocal effects are used to investigate relationships among parental homework support, effort, and achievement (see Figure 1).

##### Hypothesis 1a: Parental homework support and achievement

Consistent with related literature (Ryan and Deci, 2000; Deci and Ryan, 2008), we hypothesize that parental autonomy support and achievement would be reciprocally related. As there is little research relating to the association between content-oriented support and achievement, we leave it as a research question.

##### Hypothesis 1b: Effort and achievement

Consistent with the finding from the previous study (Marsh et al., 2016), it is hypothesized that effort would not be reciprocally associated with achievement.

##### Hypothesis 1c: Parental homework support and effort

Consistent with related literature (Dumont et al., 2014; Kikas and Silinskas, 2016), it is hypothesized that autonomy-oriented support would be reciprocally associated with effort. Similarly, as there is little research relating to the association between content-oriented support and effort, we leave it as a research question.

#### Hypothesis 2 (Robustness of Path Coefficients Over Gender)

Consistent with related literature concerning student effort and academic achievement (Deci and Ryan, 2008; Patall et al., 2008; Marsh et al., 2016), it is hypothesized that the pattern of findings concerning H_1b_ would be similar across gender. Meanwhile, given mixed results in prior research on relations between parental homework involvement and student achievement (Grolnick and Slowiaczek, 1994; Pomerantz et al., 2007; Dumont et al., 2012; Silinskas and Kikas, 2017), we do not have any hypotheses on whether H_1a_ and H_1c_ would vary across gender.

## Materials and Methods

### Participants

Participants were 336 9th graders (Mean age = 15.18 ± 0.72; 47.9% boys) from two schools in southeastern China. They were assessed two points: (a) Time 1 (October); and (b) Time 2 (June). Education level was 10.40 years (*SD* = 3.20) for father, and 9.59 years (*SD* = 3.21) for mother.

The percentages of students who did math assignments four or more days weekly were 69.7% at Time 1 and 68.2% at Time 2. The amount of time they spent on math assignments were 36.3 min (*SD* = 28.3) at Time 1 and 45.3 min (*SD* = 34.8) at Time 2. These practices are similar with other research conducted in China (Xu, 2016; Xu et al., 2017). For instance, in one study involving Chinese students in grade 8 (Xu et al., 2017), 78.5% students did math assignments four or more days weekly, with about 34 min spent on math assignments each day (*SD* = 22.0).

The current study was a part of larger international research project approved by the Institutional Review Board at University of Macau. Written informed consent was obtained from the parents/legal guardians of all non-adult participants. Several researchers administered the instrument during typical school hours, and students were given an identification number to link two different sources of data (i.e., survey data and math achievement) from Time 1 to Time 2. The identifier was removed once two waves of data were completed.

### Measures

#### Autonomy-Oriented Support

At each time point (Time 1 and 2), the same four-item scale measured autonomy-oriented support (Xu et al., 2017). These items (see Table 1) measured children’s perspectives of parental role as autonomy supportive while doing math assignment (e.g., paying attention to their ideas and encouraging their initiatives; Time 1: α = 0.90; Time 2: α = 0.90).

**Table 1:** Autonomy-oriented support, content-oriented support, and effort items.

Scales	Items	Mean (*SD*)		α (CI)	
		Time 1	Time 2	Time 1	Time 2
Autonomy-oriented support^a^	My parents encourage me to ask questions about math homework assignments.	2.67 (0.72)	2.63 (0.68)	0.90 (0.88–0.91)	0.90 (0.89–0.92)
	My parents listen to my ideas about math homework assignments.				
	My parents listen to how I would like to do math homework assignments.				
	My parents convey confidence in my ability to do with math homework assignments.				
Content-oriented support^a^	My parents often ask how they can help me with my math homework.	2.46 (0.67)	2.42 (0.66)	0.86 (0.83–0.88)	0.87 (0.85–0.90)
	My parents help me with math if I ask them.				
	My parents always help me if I get stuck with my math homework.				
	I can always ask my parents if I don’t understand something in math.				
Effort^a^	I have recently been doing my math homework to the best of my ability.	3.20 (0.61)	3.09 (0.58)	0.78 (0.73–0.82)	0.80 (0.76–0.84)
	I do my best on my math homework.				
	I always try to finish my math homework.				


*^a^Rating: 1 = Strongly disagree, 2 = Disagree, 3 = Agree, 4 = Strongly agree.*

#### Content-Oriented Support

At each wave, the same four-item scale measured content oriented support (Xu et al., 2017). These items (see Table 1) assessed the degree to which parents offered direct help on math assignments when asked by children (e.g., when children got stuck with math homework and had difficulty in figuring it out on themselves; Time 1: α = 0.86; Time 2: α = 0.87).

#### Effort

At each time point, the same three-item measured children’s effort in doing math assignments, based on relevant research on homework effort (Trautwein et al., 2006; Xu, 2016). These items (see Table 1) measured how hard students worked on these assignments (Time 1: α = 0.78; Time 2: α = 0.80).

#### Achievement

Math achievement was assessed using standardized test at each wave. The content of the test was based on national math standards in China (Li and Li, 2018). The test was designed to measure student knowledge and competence in quadratic equation, quadratic function, rotation of figures and central symmetry, circle, inverse function, trigonometric function, probability, and projection and view. Anchor items were used to allow the linkage of the two waves. At each time, participants were given 120 min to work on the test. The reliability estimate was 0.86 for Time 1, and 0.88 for Time 2.

### Data Analysis

All the analyses were implemented using Mplus (Muthén and Muthén, 1998–2012, version 7.31), where autonomy-oriented support, content-oriented support, and effort were measured by the same scales at each time point. The missing data for 11 indicators (4 autonomy-oriented support, 4 content-oriented support, and 3 effort) and one observed variable (i.e., achievement) were: Time 1 (*Mean* = 2.18%, *SD* = 0.51%), and Time 2 (*Mean* = 10.91%, *SD* = 1.66%). All models in the present investigation were based on MLR, along with FIML.

#### Measurement Invariance

Consistent with typical practices regarding multiple group invariance (Hong et al., 2003; Marsh et al., 2016), we examined gender invariance by testing configural model (baseline model), metric model (factor loading invariance), correlated uniqueness, and scalar models (intercept invariance).

#### Path Coefficient Invariance

We tested the path coefficients concerning autonomy-, content-oriented support, effort, and achievement from Time 1 to Time 2. Also included in the path model were paths regarding the same construct from Time 1 to Time 2. For example, Time 2 autonomy-oriented support was predicted by Time 2 content-oriented support, effort, and achievement, but also by Time 1 autonomy-oriented support). Hence, to test invariance of path coefficients, 16 paths were constrained equal over gender (Figure 1; 12 cross paths and 4 horizontal paths).

#### Goodness of Fit

We applied a number of goodness-of-fit indexes: (a) comparative fit index (CFI) near 0.95 (Hu and Bentler, 1999), (b) standardized root mean square residual (SRMR) ≤ 0.08 (Hu and Bentler, 1999), and (c) root mean square error of approximation (RMSEA) ≤ 0.06 (MacCallum et al., 1996), and Additionally, we applied the following recommendations for multigroup invariance testing; there is a support for more parsimonious model when ΔCFI < 0.01 and ΔRMESA < 0.015 (Cheung and Rensvold, 2002; Chen, 2007).

## Results

The findings of the current investigation are presented into two sections. Section 1 centers on the factor structure represented the 22 indicators and the 2 observed variables. Part 2 investigates the models of reciprocal influences of autonomy-, content-oriented support, effort, and achievement using two waves of data.

### The Factor Structure

We tested the factor structure invariance over gender (161 boys vs. 175 girls), by examining the following models: configural, metric, correlated uniqueness, and scalar (see Table 2). Overall, these models produced good fits (e.g., all CFIs ≥ 0.945). In addition, the fit of the most constrained Model 4 (scalar) was good (CFI = 0.945; RMSEA = 0.057; SRMR = 0.066), which hardly differed from that of the least-constrained Model 1 (configural; ΔCFI = 0.007, ΔRMESA = 0.001). Thus, these findings supported the invariance of factor structure for males and females.

**Table 2:** Tests for gender invariance: summary of goodness-of-fit statistics.

Invariance models	MLRχ^2^	df	RMSEA	RMSEA 90% CI	CFI	SRMR
(1) Configural (baseline)	656.347	430	0.056	0.047–0.064	0.952	0.054
(2) Metric (factor loading)	669.268	446	0.055	0.046–0.063	0.952	0.058
(3) Correlated uniqueness	701.813	457	0.056	0.048–0.065	0.948	0.063
(4) Scalar (intercept)	728.449	473	0.057	0.048–0.065	0.945	0.066
(5) Path coefficient	744.322	489	0.056	0.048–0.064	0.945	0.073


*RMSEA, root mean square error of approximation; CFI, Comparative Fit Index; SRMR, standard root mean squared residual.*

As displayed in Table 3, the standardized factor loadings for each wave were quite large. Across both time points, the factor loadings ranged 0.743–0.889 for autonomy-oriented support, 0.719–0.875 for content-oriented support, and 0.713–0.821 for effort.

**Table 3:** Standardized factor loadings.

Variables	Time 1 constructs				Time 2 constructs			
	AO	CO	EF	Test	AO	CO	EF	Test
T1AO1	0.818							
T1AO2	0.889							
TIAO3	0.866							
TIAO4	0.743							
T1CO1		0.745						
T1CO2		0.719						
T1CO3		0.858						
T1CO4		0.794						
T1EF1			0.713					
T1EF2			0.789					
T1EF3			0.708					
T1TEST				1				
T2AO1					0.849			
T2AO2					0.873			
T2AO3					0.869			
T2AO4					0.757			
T2CO1						0.767		
T2CO2						0.725		
T2CO3						0.875		
T2CO4						0.828		
T2EF1							0.732	
T2EF2							0.821	
T2EF3							0.731	
T2TEST								1


*Each variable was assigned a label that identifies the Time (T1 or T2), the construct (AO, autonomy-oriented support; CO, content-oriented support; EF, effort; and Test, math achievement), and for the multiple indicators of each latent construct, and the item number. In both waves, autonomy-oriented support was measured with the same four items (AO1–AO4), content-oriented support was measured with the same four items (CO1–CO4), and effort was measured with the same three items (EF1–EF3), whereas test was based on a single score for each wave.*

Within each of the two waves, there were large positive correlations between autonomy-oriented support and content-oriented support (0.56; see Table 4). Additionally, there were medium to large positive correlations between autonomy-oriented support and effort (0.27–0.37) and between effort and achievement (0.26–0.47). Furthermore, there were small positive correlations between autonomy-oriented support and achievement (0.17–0.23) and between content-oriented support and effort (0.16–0.17). Finally, there were non-significant to significant small negative correlations between content-oriented support and achievement (-0.07 – -0.12).

**Table 4:** Factor correlations.

	Time 1 constructs				Time 2 constructs			
	AO	CO	EF	Test	AO	CO	EF	Test
**Time 1**
AO	1.000							
CO	0.56***	1.000						
EF	0.27***	0.16*	1.000					
Test	0.17**	–0.12*	0.47***	1.000				
**Time 2**
AO	0.64***	0.43***	0.32***	0.18**	1.000			
CO	0.39***	0.65***	0.12	–0.08	0.56***	1.000		
EF	0.23***	0.27***	0.67***	0.25***	0.37***	0.17*	1.000	
Test	0.21***	–0.11	0.48***	0.91***	0.23***	–0.07	0.26***	1.000


*AO, autonomy-oriented support; CO, content-oriented support; EF, effort; and Test, achievement. ^∗^*p* < 0.05. ^∗∗^*p* < 0.01. ^∗∗∗^*p* < 0.001.*

### Reciprocal Effects Among Parental Homework Support, Effort, and Achievement

We further tested the structural path invariance over gender. As shown in Table 2, data showed a good fit (CFI = 0.945; SRMR = 0.073; RMSEA = 0.056; 90% CI [0.048 -0.064]), which hardly differed from that of Model 4 (ΔCFI < 0.001, ΔRMESA = 0.001). These findings supported the structural path invariance over gender.

#### Parental Homework Support and Achievement

There were no reciprocal effects between autonomy-oriented support and achievement (see Table 5). Not unexpectedly, the largest influence of T1 autonomy-oriented support was on T2 autonomy-oriented support (β = 0.535, *p* < 0.001). The influence of T1 autonomy-oriented support was statistically significant for T2 achievement (β = 0.079, *p* < 0.01), after controlling the effects of other T1 measures (content-oriented support, effort, and achievement). However, T1 achievement had a non-significant influence on T2 autonomy-oriented support (β = 0.031, *p* > 0.05).

**Table 5:** Path coefficients for models of reciprocal effects among autonomy-oriented support (AO), content-oriented support (CO), effort (EF), and test (math achievement).

Dependent variable/ Independent variable	Male		Female		Overall	
	Path coefficient	*SE*	Path coefficient	*SE*	Path coefficient	*SE*
**T2AO**					
T1AO	0.547***	0.074	0.524***	0.074	0.535***	0.072
T1ICO	0.106	0.069	0.100	0.065	0.105	0.066
T1EF	0.131*	0.060	0.108*	0.052	0.144*	0.060
T1TEST	0.035	0.053	0.040	0.060	0.031	0.057
**T2CO**					
T1AO	0.024	0.081	0.021	0.072	0.042	0.076
T1CO	0.668***	0.086	0.579***	0.070	0.621***	0.070
T1EF	0.021	0.071	0.016	0.053	0.012	0.064
T1TEST	0.021	0.055	0.022	0.058	0.021	0.058
**T2EF**					
T1AO	0.033	0.070	0.037	0.079	0.047	0.072
T1CO	0.172*	0.075	0.191*	0.081	0.178*	0.076
T1EF	0.662***	0.087	0.639***	0.073	0.668***	0.072
T1TEST	0.019	0.061	0.026	0.083	0.032	0.070
**T2TEST**					
T1AO	0.093**	0.033	0.079**	0.029	0.079**	0.030
T1CO	0.076*	0.032	0.064*	0.026	0.066*	0.027
T1EF	0.088*	0.040	0.065*	0.031	0.077*	0.035
T1TEST	0.840***	0.031	0.859***	0.030	0.851***	0.029


*^∗^p < 0.05. ^∗∗^*p* < 0.01. ^∗∗∗^*p* < 0.001.*

Additionally, there were no reciprocal influences of content-oriented support and achievement. Whereas T1 content-oriented support had a substantial influence on T2 content-oriented support (β = 0.621, *p* < 0.001), it had a negative influence on T2 achievement (β = -0.066, *p* < 0.05). Meanwhile, T1 achievement had a non-significant influence on T2 content-oriented support (β = -0.021, *p* > 0.05).

#### Effort and Achievement

There was no support for reciprocal influences of effort and achievement. T1 effort had a statistically significant effect on T2 achievement (β = 0.077, *p* < 0.05). However, T1 achievement had a non-significant influence on T2 effort (β = -0.032, *p* > 0.05).

#### Parental Homework Support and Effort

There were no reciprocal effects between autonomy-oriented support and effort. T1 effort had a significant effect on T2 autonomy-oriented support (β = 0.144, *p* < 0.05), after controlling the effects of other T1 measures (autonomy-, content-oriented support, and achievement). However, T1 autonomy-oriented support did not have a significant influence on T2 effort (β = -0.047, *p* > 0.05).

Additionally, there were no reciprocal influences of content-oriented support and effort. T1 content-oriented support had a significant effect on T2 effort (β = 0.178, *p* < 0.05), after controlling the effects of other T1 measures (autonomy-oriented support, effort, and achievement). However, the path from T1 effort to T2 content-oriented support was not significant (β = 0.012, *p* > 0.05).

## Discussion

We examined the reciprocal influences of parental homework support, effort, and achievement over two time points concerning math homework. Informed by self-determination theory (Deci and Ryan, 2008; Ryan et al., 2016) and Grolnick and Slowiaczek (1994) two models concerning the effects of parent involvement, we examined several hypotheses, some extending previous research, while others providing seemingly intriguing theoretical perspectives.

### Parental Homework Support and Achievement

**FIGURE 2:**
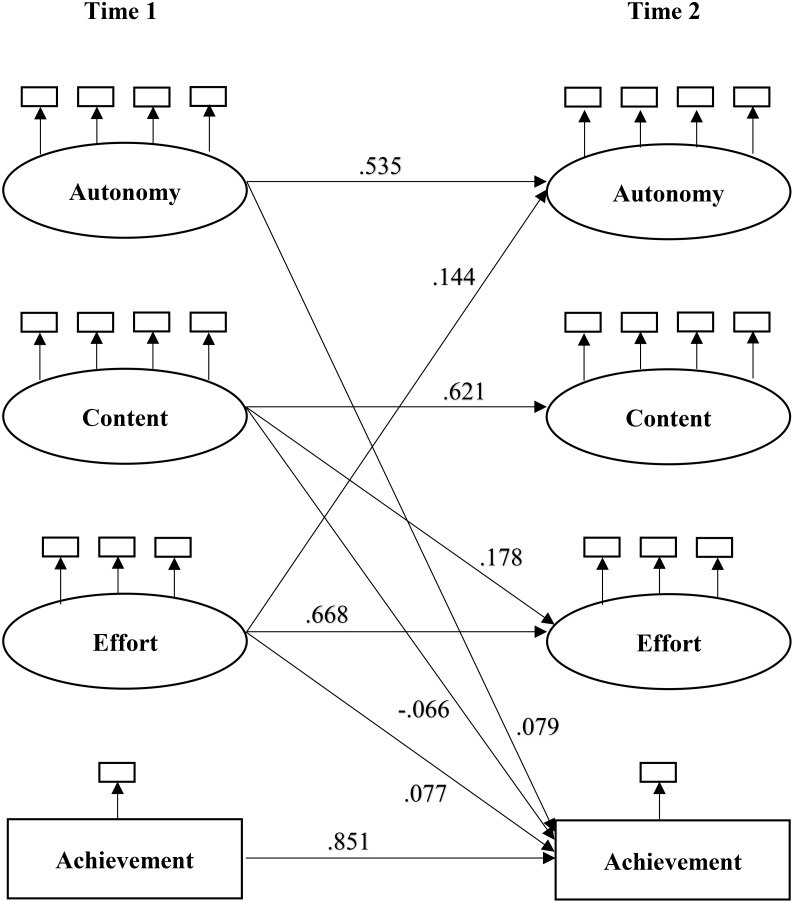
Structural equation paths relating Time 1 (T1) to Time (T2). Only statistically significant paths are displayed.

There was an asymmetrical pattern of reciprocal influences of autonomy-oriented support and achievement: prior higher autonomy-oriented support led to higher subsequent achievement, yet prior achievement was unrelated to subsequent autonomy-oriented support (see Figure 2). Furthermore, there was an asymmetrical pattern of reciprocal influences of content-oriented support and achievement: higher prior content-oriented support resulted in lower subsequent achievement, yet prior achievement was unrelated to subsequent content-oriented support.

The finding that higher prior autonomy-oriented support led to higher subsequent achievement is congruent with previous research concerning the role of autonomy support on student learning (e.g., Deci and Ryan, 2008), with homework in particular (Xu et al., 2017). Meanwhile, how do we interpret the result that prior content-oriented support had a negative effect on subsequent achievement? As compared with autonomy support, direct help from parents is viewed as more controlling particularly when children do not ask for help; it may result in a decreased sense of autonomy over time (Pomerantz et al., 2007). As content-oriented support in our study is conceptualized as the extent to which parents provide direct help on homework when asked by children, our study suggests that parental help may backfire even when asked by children. One possible explanation is that content-oriented support (i.e., even when asked by children) may lead to a sense of incompetence for children (e.g., implying that they could not solve math problems independently), which may in turn undermine subsequent achievement. Another explanation is that when asked by children for content-oriented support, many parents may find it difficult to withdraw their support as children become more competent and are well on their own. This explanation is somewhat substantiated by one observation drawn from their research synthesis on parental homework involvement that “as students reach adolescence, it may be important that parents gradually withdraw from the homework process and shift their involvement more to support of the child’s own autonomous efforts.” (Patall et al., 2008, p. 1089). Taken together, it could be argued that the present study extends prior research in the following way. That is, while consistent with previous studies that direct support has detrimental effect on student achievement (e.g., Veas et al., 2015; Fernández-Alonso et al., 2017), content-oriented support – even when asked by children – is likely to be controlling (e.g., in terms of what it means to children for homework assignments at hand, and what it means to parents for homework involvement over time).

### Effort and Achievement

There was an asymmetrical pattern of reciprocal influences of effort and achievement: higher prior effort led to higher subsequent achievement, while prior achievement was unrelated to subsequent effort. Our findings were not congruent with the prior finding (Marsh et al., 2016) concerning the lack of any support for reciprocal influences of effort and achievement (assessed by standardized test scores). One likely explanation is that Chinese culture emphasize the important role of effort in student achievement (Rao et al., 2000; Li, 2002). Thus, it makes logical sense that prior effort may have a more pronounced influence on subsequent academic achievement for Chinese students in particular.

### Parental Homework Support and Effort

There was an asymmetrical pattern of reciprocal influences of autonomy-oriented support and effort: higher prior effort led to higher subsequent autonomy-oriented support, yet prior autonomy-oriented support was unrelated to subsequent effort. There was also an asymmetrical pattern of reciprocal influences of content-oriented support and effort: higher prior content-oriented support led to higher subsequent effort, yet prior effort was unrelated to subsequent content-oriented support.

These results provide partial empirical support to the commonly held assumption that whereas parents’ behavior (e.g., parental support) influences the child’s school-related behavior, the characteristics of the child (e.g., effort) can also influence parents’ behavior (Grolnick and Slowiaczek, 1994; Raftery et al., 2012; Dumont et al., 2014; Kikas and Silinskas, 2016). On the other hand, our findings provide a more nuanced picture, in that the relations between different forms of parental homework support and homework effort were not asymmetrical (i.e., higher prior content-oriented support led to higher subsequent effort, whereas higher prior effort led to higher subsequent autonomy-oriented support).

How do we interpret the results that prior effort had a positive effect on subsequent autonomy-oriented support (yet unrelated to subsequent content-oriented support)? It seems logical that as children put more efforts in doing math homework, parents are more likely to pay attention to children’s idea, encourage them to solve math problems by themselves, and express confidence in their capacities in following through math assignments. Meanwhile, as content-oriented support is referred to the degree to which parents offered direct help on homework when requested by children, putting more effort in homework may lead to less request for content-oriented support. Yet, more homework effort may also lead to more request for content-oriented support, as “students who exert greater task-oriented effort do not refrain from seeking needed help” (Karabenick and Knapp, 1991, p. 224). This observation is, to some degree, substantiated by zero-order correlation from the study by Skaalvik and Skaalvik (2013), in which effort (e.g., “I always do my homework.”) was positively associated with certain help-seeking behavior (e.g., “If there is something I do not understand at school, I ask the teacher for help”).

In addition, how do we interpret the results that prior content-oriented support (but not prior autonomy-oriented support) had a positive influence on subsequent effort? The finding concerning content-oriented support was consistent with Trautwein et al. (2006) in that parental provision of help (which is comparable to content-oriented support) was positively associated with homework effort. Yet, the finding concerning autonomy-oriented support is not consistent with related literature that autonomy support can promote task-oriented effort (Pomerantz et al., 2007; Deci and Ryan, 2008). One possible explanation is that in an achievement domain such as a math that requires more effort (Marsh et al., 2016), content-oriented support (compared with autonomy-oriented support) may play a more important role in promoting student effort in following through math homework. In other words, as working on math assignments in particular is viewed as a considerable challenge for many students (e.g., math anxiety; Else-Quest et al., 2008), it makes sense that students need content-oriented support (i.e., more than autonomy-oriented support) to enable them to exhibit more effort in completing math assignments in the face of various obstacles and difficulties (e.g., when they get stuck with math homework). This is further consistent with qualitative findings from US secondary students that content-oriented support (e.g., content-related parental assistance concerning algebra and geometry) had a positive effect on students’ effort to complete their homework (Martinez, 2011).

### Strengths, Limitations, and Directions for Further Research

Our investigation represents a significant advance over prior research on parental homework involvement by using models of reciprocal effects to examine relationships among parental homework support, effort, and achievement. Even though these are not causal effects, they permit a more robust examination of the relationships among these variables (e.g., concerning the direction of relationships; Selig and Little, 2012). Additionally, our study concerning the structural path invariance imply that our results are applicable over gender. As the fulfillment of measurement invariance is a prerequisite for meaningful and substantive cross-group mean comparisons, our current study extends prior research on parental homework involvement.

Specifically, as no prior studies that have studied the relationships among these constructs using models of reciprocal effects, our findings provide new insights concerning the role of prior parental homework support (i.e., autonomy-oriented support vs. content-oriented support) on subsequent homework effort and achievement, as well as the role of prior homework effort on subsequent autonomy-oriented support. Taken together, these findings extends our understanding of parental homework support, suggesting the need to differentiate these two types of parental homework support in future investigation.

The effect size in the present investigation were small. However, they represent longitudinal relationships. Indeed, small effect size are common, but not trivial while examining longitudinal changes (Adachi and Willoughby, 2015; Willoughby et al., 2015), as they reflect an ongoing process of cumulative and addictive effects.

As our study was based on students from two schools during one school, it would be important to replicate our findings using a representative sample of students in other settings over a longer period of time. Although our investigation incorporated standardized achievement tests to measure math achievement, we assessed parental homework involvement and effort using self-report measures. Hence, there is a need to include multiple sources in further research (e.g., direct observation or parent reports). Meanwhile, like other researchers (Trautwein et al., 2012; Dumont et al., 2014), given the focus on children’s perceptions of parental homework support, children’s reports is the most appropriate and valid indicator of how they perceive their parental homework support as well as their own effort in the homework process (even if other sources may offer alternative perspectives).

As this is the first study, to our knowledge, to investigate reciprocal relations among parental homework support, effort, and achievement, it is important to continue this line of research in other countries, as cultural values may affect the relations among these constructs (e.g., cultural norms concerning autonomy, effort, academic learning, and role of parents in the homework process; Rao et al., 2000; Ryan and Deci, 2000; Xu et al., 2017). It would also be important to pursue this line of investigation at different development stages, as (a) the role of parental homework involvement on academic achievement was found to be moderated by school level (Patall et al., 2008), and as (b) parental involvement declines as children move from elementary to secondary school (Gonida and Cortina, 2014). Additionally, as parental rule-setting (Patall et al., 2008) or perceived parental structure (Dumont et al., 2014) were positively related to homework effort and achievement for younger students (grades 2–7), it would be intriguing to reframe this form of parental homework involvement as structure-oriented support and to incorporate it in future research on parental homework support at elementary school level in particular (i.e., along with autonomy- and content-oriented support).

In addition, there is a need to study reciprocal influences among parental homework support, effort, and achievement in different achievement areas (e.g., science), as (a) some achievement domains do not require similar degree of effort as math (Marsh et al., 2016), as (b) parental homework involvement may play out differently in math as compared with other achievement domains (Patall et al., 2008), and as (c) our results suggest that content-oriented support plays a more prominent role in promoting student effort in following through math assignments.

## Conclusion

In conclusion, by using models of reciprocal effects among parental homework support (autonomy- and content-oriented support), effort, and achievement, our present study shed new insights into the relationships among these constructs – a promising line of investigation that has been inadequately conceptualized and studied in last several decades.

## Ethics Statement

This research was carried out in according with the recommendations of the University of Macau. Written informed consent was obtained from the parents/legal guardians in accordance with the Declaration of Helsinki.

## Author Contributions

JX contributed to the conception and the design of the work. JD was responsible for data collection. SW, HR, and AC contributed to literature review and several sections of writing. JX and JD contributed to manuscript write up and revision.

## Conflict of Interest Statement

The authors declare that the research was conducted in the absence of any commercial or financial relationships that could be construed as a potential conflict of interest.
